# Correction to: Role of financial incentives in family planning services in India: a qualitative study

**DOI:** 10.1186/s12913-021-07091-y

**Published:** 2021-12-02

**Authors:** Kamlesh Lalchandani, Aditi Gupta, Ashish Srivastava, Gulnoza Usmanova, Ashwarya Maadam, Bulbul Sood

**Affiliations:** Jhpiego - an affiliate of Johns Hopkins University, New Delhi, 110020 India

**Correction to:** ***BMC Health Serv Res*** **21, 905 (2021)**

**https://doi.org/10.1186/s12913-021-06799-1**

Following publication of the original article [1], multiple language errors were identified because the typesetter didn’t implement these corrections during production. The changes have been highlighted with **corrections** and shown in Additional file 1.

The original article [1] has been corrected.

## Supplementary Information

**Additional file 1.**
