# Supplementary material for: Role of financial incentives in family planning services in India: a qualitative study
Source: BMC Health Serv Res. 2021 Sep 3;21:905. doi: 10.1186/s12913-021-06799-1 (PMC8414850; doi:10.1186/s12913-021-06799-1)
Supplement: Supplementary file 1 — Additional file 1. [file 12913_2021_6799_MOESM1_ESM.docx]

**Additional File 1**

**Role of Incentives in the Uptake and Service Provisioning for Family Planning in Jharkhand**

**Key Informant TOOLS FOR service providers (ANMs/Nurses)**

**Key Themes to Explore**

- Her role and motivation in FP service provision
- Challenges in providing quality services
- The role of incentives for her in providing the services
- Her opinion on the status and motivation of uptake of FP services among women
- Her view on the role of incentives for clients and the possibilities of alternatives

**About the ANM/ Nurse**

1. Can you tell me something about yourself (probe: how long in service, education, etc)?

**About FP Services**

1. What is the general status of uptake of FP services among eligible couples in your facility/area?

(Probe: percentage uptake, popular methods, factors which influence uptake)

1. What is your main role in providing FP services? (probe: motivation; follow-up; probe by method)
2. What kind of training and capacity building inputs have you received for providing the FP services
3. In the last one year, how many women have you provided services to and for what methods? (probe by different methods- including IUCD, Oral Pills)
4. What are some of the key challenges that you experience in the provision of FP services?

**Incentives (Clients)**

1. What are the factors which generally motivate a client to take up a FP method? (Probe: rank in order of importance)
2. What are the reasons for high uptake of FS and PPIUCD compared to the other methods?
3. To what extent and how do incentives motivate a client for taking up a FP method? (Probe: Variation across different sections)
4. How does the varying amount of incentives for different methods influence client’s decision of adopting a method?
5. How will FP uptake be impacted if incentive amounts are changed? (Probe: impact of abolishing, decreasing, or increasing the amount for clients, motivators, and service providers
6. What difference will it make if monetary incentives for clients are replaced with non-monetary incentives or commodities like cereals, milk powder etc.? (Probe: which is better, other possible non-monetary incentives)
7. What difference will it make if monetary incentives for clients are replaced with improved quality of services like better cleanliness at the health facilities? (Probe: which is better, possible ways in which quality can be further improved, non-monetary incentives vs improved quality of services)

**Incentive (Self)**

1. How much incentives have you got from FP services in last one year? What part of your total earning from service provision was it?
2. What is the process and procedure for claiming the incentives? (probe: when do the file claims; process, the process for follow -up services etc.)
3. What has been your challenges in getting the incentives and how does that affect your service provision? (time to disbursement, mode of disbursement, delays in payments etc.,)
4. To what extent do incentives motivate you for service provision and why?
5. How would your service provision be affected if there were no incentives for the FP methods?
6. What difference will it make if monetary incentives for providers are replaced with non-monetary incentives like awards or recognition? (Probe: which is better? Other possible non-monetary incentives)
7. What difference will it make if provider-based incentives are replaced with facility-based incentives? (Probe: Which are better and possible ways of giving facility-based incentives)
8. What would be your suggestions for the provision of incentives in family planning? (Probe: Improvement in quality of FP services vs incentives)

**Other Issues**

1. Would you like to add anything else?

**Role of Incentives in the Uptake and Service Provisioning for Family Planning in Jharkhand**

**Key Informant Interview Tool For Doctors**

**Key Themes to be Explored**

- The status of service provision for FP in the different facilities he/she is attached to
- The key challenges that he sees in the service provision for FP in the area?
- The factors that influences uptake of service provision and uptake of FP methods by client
- The role of incentives in service provision and uptake of FP and the possibilities of alternatives

**About the Doctor**

1. Can you tell me something about yourself (probe: how long in service, posting etc. his role in provision for FP services)?
2. What all FP services do you provide and which all facilities (probe: FS/NSV/ PPIUCD; facilities DH/CHC/ others; frequency)

**About FP Services**

1. What is the general status of uptake of FP services? (Probe: percentage uptake by method, popular methods etc.) factors which influence uptake)
2. What are the factors that influence the uptake of FP methods and services? (probe: factors that motivate a client to take up a FP method; rank in order of importance; administrative and other issues)
3. What are the reasons for high uptake of FS and PPIUCD compared to the other methods?
4. What are some of the key challenges in the provision of FP services in the area (probe: lack of doctors; facilities; service providers; seasonality; others)
5. What are the main challenges you face in providing the FP services and why?

**Incentives (Self)**

1. What kind of incentives do you get for the different services that you provide? (probe by method)
2. What are some of the challenges you have faced with claiming and receiving incentives? (probe: paper work; delays in payment; structure of disbursement)
3. In the last year what has been the total amount in incentives that you received for FP services rendered?
4. How important are incentives from FP as a source of earning in comparison to the salary you receive? (or other sources of incentives)
5. To what extent and how do incentives motivate you for providing FP related services?
6. What if incentives for doctors was stopped?
7. What kind of incentives do you think will incentivize doctors for better service provision for FP? (probe: better pay; better facilities for personal life; facility-based incentives etc.)

**Incentives (clients)**

1. To what extent and how do incentives motivate a client for taking up a FP method? (Probe: Variation across different sections; variation by methods-including non-incentivized methods)
2. How will FP uptake be impacted if incentive amounts are changed? (Probe: impact of abolishing, decreasing, or increasing the amount)
3. What difference will it make if monetary incentives for clients are replaced with non-monetary incentives or commodities like cereals, milk powder etc. or with improved quality of services like better cleanliness at the health facilities? (Probe: which is better, other possible non-monetary incentives)

**Incentives (other service providers)**

1. In your opinion how does provision of incentives influence quality of FP services of other service providers (ASHA/ANM/Nurses)?
2. How does varying amount of incentives for different methods influence a provider’s services and communication with client on FP methods? (probe for ASHA/ANM/Nurse)
3. What difference will it make to service providers if monetary incentives are replaced with non-monetary incentives like awards or recognition facility -based incentives? (Probe: which is better? Other possible non-monetary incentives, effect on different cadre of health service providers; better and possible ways of giving facility-based incentives)
4. What would be your suggestions for the provision of incentives in family planning to service providers and clients? (Probe: Improvement in quality of FP services vs incentives)

**Others**

1. Would you like to add anything else?

**Role of Incentives in the Uptake and Service Provisioning for Family Planning in Jharkhand**

**Key Informant Interview Tool FOR Government Health Officials**

**Key Themes to be Explored**

- Key factors and issues that affect the FP service provision and uptake
- The role on incentives in the service provision and uptake of FP services
- The alternatives to incentives and its potential impact on service provision and uptake of FP services
- Future and suggestions for improving the services and uptake for FP

**About the Official**

1. Could you tell us something about yourself **(**probe**:** what service; how long in the position, experience with FP service sector)

**FP services and Uptake**

1. What is the general status of uptake of FP services? (Probe: percentage uptake by method, popular methods etc.- for districts probe for Block wise ranking of FP service performance; for state district wise performance)
2. How is the information on the uptake of different FP methods maintained and monitored? (probe: levels, methods; validity; challenges)
3. What are the factors that influence the uptake of FP methods and services? (probe: factors that motivate a client to take up a FP method; rank in order of importance; administrative and other issues; training)
4. What are the reasons for high uptake of FS and PPIUCD compared to the other methods?
5. What are some of the key challenges in the provision of FP services in the area (probe: budget flow; policy issues, vacancies; infrastructure; lack of doctors)?
6. What were some of the reasons behind considering your district / state under MPV?
7. What are your suggestions to improve the uptake and services for FP?

**Incentives (Client)**

1. To what extent and how do incentives motivate a client for taking up a FP method? (Probe: Variation across different sections; variation by methods-including non-incentivised methods)
2. How will FP uptake be impacted if incentive amounts are changed? (Probe: impact of abolishing, decreasing, or increasing the amount)
3. What difference will it make if monetary incentives for clients are replaced with non-monetary incentives or commodities like cereals, milk powder etc. or with improved quality of services like better cleanliness at the health facilities? (Probe: which is better, other possible non-monetary incentives)

**Incentives (other service providers)**

1. In your opinion how does provision of incentives influence quality of FP services of other service providers (Doctors/ ASHA/ANM/Nurses)?
2. How does varying amount of incentives for different methods influence a provider’s services and communication with client on FP methods? (probe for Doctors/ ASHA/ANM/Nurse)
3. What difference will it make to service providers if monetary incentives are replaced with non-monetary incentives like awards or recognition facility -based incentives? (Probe: which is better? Other possible non-monetary incentives, effect on different cadre of health service providers; better and possible ways of giving facility-based incentives)
4. What would be your suggestions for the provision of incentives in family planning to service providers and clients towards improving uptake and service? (Probe: Improvement in quality of FP services vs incentives)

**General Questions on Incentives**

1. What are some of the challenges in providing the incentives to the clients and the service providers? (probe: delay in payment; availability of resources; etc)?
2. How could the challenges identified above be addressed?
3. What are the reasons behind revising the incentive structures for some methods under MPV?
4. What are some of the new measures being taken to improve planning services and uptake?
5. Could the resources available for incentives be better used to improve uptake and quality service of FP?

**Others**

1. Would you like to add anything else?

**Role of Incentives in the Uptake and Service Provisioning for Family Planning in Jharkhand**

**IDI Tool for FEMALE Clients**

**Themes to be explored**

- Basic socio-economic context of the person
- knowledge of the FP method she has opted for
- Decision-making process in opting for the FP method
- Her experiences of using the FP method and services
- Role and use of Incentive in the uptake of the method
- Her opinion on monetary and non-monetary incentives
- Her suggestions for improving her experience of the service and use of the FP Method.

**About the Client**

1. Can you tell me something about yourself (probe: family/social category/ work/ education etc.)?

**About the FP Method and Service**

1. Since when have you been using this method and where did you get the services for this? (probe: how long back/ which health centre etc.)
2. What has been your experience in using this method so far? (Probe: pain/discomfort/removal/visit to any govt or private service provider; follow-up services if any))
3. If any problem was experienced, how did you seek redressal? (probe: where did she go and what was her experience receiving the redress)

**Decision Making**

1. How and why did you decide to go in for family planning? (probe: what and who motivated her/ others involved in taking this decision)
2. Can you tell me something about the FP method you use? (probe: her knowledge of advantages/ disadvantages/ side effects /how to manage it etc.)
3. How did you decide to use this method? (probe: reasons for choosing/ role of family and/or service providers and/or other involved)
4. What have you been told about using this method and by whom?
5. Who all have you approached to get more information about this method?
6. What other methods did you have information about and from whom?
7. What were the reasons for not opting for the other methods available?

**Experience with service received**

1. Can you explain in detail about the services you received for this method? (probe: access and support received to access to centre; counselling; quality of service; quality of service; post care and follow up; level of satisfaction)
2. What according to you are some of the aspects of the service that could have been improved or better?

**Incentives**

1. How much money did you get for adopting the method?
2. What was your experience of getting the money? (probe: process; ease; delay; paperwork etc.)
3. Why do you think the government provides money for taking up the FP method?
4. What did you use the money for?
5. How much did the fact that you would get money in adopting this method influence your decision to opt for this method?
6. What if the monetary incentive was not given?
7. If the government decided not to give the money but something else instead to the FP client – what would you want the government to give and why?
8. In your view what difference will it make if monetary incentives are replaced with non-monetary incentives or commodities like cereals, milk powder etc.? (also probe: for improved quality of care instead of monetary incentives)

**General Opinion /perception on Incentives and FP**

1. In your view, what are some of the reasons for women opt for FP methods?
2. How and where do women get information on FP methods generally? (probe: diff sources/type and quality of information)
3. In your view, to what extent do incentives motivate a client to take up a up a FP method?
4. How do you think the uptake of FP amongst women will be impacted if monetary incentives are stopped?

**Other Issues**

1. Would you like to add anything else?

**Role of Incentives in the Uptake and Service Provisioning for Family Planning in Jharkhand**

**Key Informant Interview Tool -ASHA**

**Key Themes to Explore**

- To understand her role, training and capacities in motivating clients for FP
- To understand the process, they follow on the ground for the different methods (how it varies from prescribed protocol) -including motivation and follow-up services
- To understand what motivates women to take up the FP methods
- To understand the importance incentives for women in decision making and uptake of a method
- To understand the role of incentives and its importance of incentives ASHAs in promoting and providing services for FP methods.

**About the ASHA**

1. Can you tell me something about yourself (probe: how long in service, education, family etc).

**About FP Services**

1. What is the general status of uptake of FP services among eligible couples in your facility/area? (Probe: percentage uptake, popular methods)
2. What is your main role in providing FP services? (probe: motivation; follow-up)
3. What trainings or other capacity building inputs have you received or receive for carrying out your role in FP services? (probe: training on specific methods and their side effects, technical trainings/ source of training)
4. Can you explain in detail what is the process involved in executing your role? (probe: maintaining register of eligible couples/ outreach /motivation /accompanying for services follow-up etc.)
5. How do you motivate women FP services? What information do you provide (what options does she offer and what information about the method she offers)?
6. Where and how do you access information related to FP methods and services?
7. How many women have you motivated in the last one year/six months and for what methods? (probe: if mainly FS and PPIUCD then ask why these only and no other methods)
8. What FP methods do women mostly opt for and why?
9. What challenges do you face in motivating women for uptake for different methods? (probe: by methods; outreach to women; follow-up; burden of work; lack of knowledge)
10. What are some of the challenges you face in your role in providing FP services?
11. What factors generally motivate a client to take up a FP method?

**Incentives (in concern with the particular Client)**

1. Was the client self-motivated or you motivated her for adopting FP method?
2. What method did you counsel her for and why?
3. Why did the client use the particular method?
4. In your view, to what extent had incentive motivated the client to take up a FP method?
5. How do you think the uptake of FP amongst women will be impacted if monetary incentives are stopped?
6. In your view what difference will it make if monetary incentives are replaced with non-monetary incentives or commodities like cereals, milk powder etc.?
7. In your view how would improving the quality of services but not offering the incentive impact the uptake of FP services?
8. What are the reasons for high uptake of FS and PPIUCD compared to the other methods?

**Incentives (self)**

1. How much incentives have you got from FP services in last one year? What part of your total earning from service provision was it?
2. What is the process and procedure for claiming the incentives? (probe: when do the file claims ; process, the process for follow -up services etc.)
3. What has been your challenges in getting the incentives and how does that affect your service provision? (time to disbursement, mode of disbursement, delays in payments etc., payments for follow-up)
4. To what extent do incentives motivate you for service provision and why?
5. How would your service provision be affected if there were no incentives for the FP methods?
6. What difference will it make if monetary incentives for providers are replaced with non-monetary incentives like awards or recognition? (Probe: which is better? Other possible non-monetary incentives)

**Other Issues**

1. Would you like to add anything else?
