# Supplementary material for: Correction to: Role of financial incentives in family planning services in India: a qualitative study
Source: BMC Health Serv Res. 2021 Dec 2;21:1291. doi: 10.1186/s12913-021-07091-y (PMC8638325; doi:10.1186/s12913-021-07091-y)
Supplement: Supplementary file 1 — Additional file 1. [file 12913_2021_7091_MOESM1_ESM.pdf]

BMC Health Services Research

RESEARCH ARTICLE Open Access

Role of financial incentives in family planning services in India: a qualitative study

Kamlesh Lalchandani, Aditi Gupta*, Ashish Srivastava, Gulnoza Usmanova, Ashwarya Maadam and Bulbul Sood

Abstract

Background: In an effort to encourage Family Planning (FP) adoption, since 1952, the Government of India has been implementing various centrally sponsored schemes that offer financial incentives (FIs) to acceptors as well as service providers, for services related to certain FP methods. However, understanding of the role of FIs on uptake of FP services, and the quality of FP services provided, is limited and mixed.

Methods: A qualitative descriptive study was conducted in Chatra and Palamu districts of Jharkhand state. A total of 64 interviews involving multiple stakeholders were conducted. The stakeholders included recent FP acceptors or clients, FP service providers of public health facilities including community health activists (ASHAs), government health officials managing FP programs at the district and state level, and members of development partners supporting FP programs in Jharkhand. Data analysis included both inductive and deductive strategies. It was done using the software Atlas ti version 8.

Results: It has emerged that there is a strong felt need for FP among majority of the clients, and FIs may be a motivator for uptake of FP methods only among those belonging to the lower socio economic strata. For ASHAs, FI is the primary motivator for providing FP related services. There may be a tendency among them and nurses to promote methods which have more financial incentives linked with them. There are mixed opinions on discontinuing FIs for clients or replacing them with non-financial incentives. Delays in payment of FIs to both clients and the ASHAs is a common issue and adversely effects the program.

Conclusion: FIs for clients have limited influence on their decision to take up a FP method while different amounts of FIs for ASHAs and nurses, linked with different FP methods, may be influencing their service provision. More research is needed to determine the effect of discontinuing FP related FIs for clients.

Keywords: Family planning, Incentives, Qualitative study, India

Background

Financial incentives (FI) serve as one of the key factors contributing towards increasing access and quality of healthcare services globally [1]. They are seen as extrinsic factors of motivation both, for the healthcare providers as well as the clients [1]. One of the popular approaches which has been widespread in countries such as the United States, Australia, Germany, Canada, UK is 'Pay for performance' where, an explicit amount of financial incentive is paid to the healthcare provider based on special performance measurements [2]. When offered as a reward for clinicians' effective performance, FI effectively decreases the number of medical errors and enables fewer events of malpractice [3]. Whilst for patients, FI is believed to be an encouraging factor influencing the uptake and adherence to the prescribed services [1].

* Correspondence: dr.aditigupta86@yahoo.com
Jhpiego - an affiliate of Johns Hopkins University, New Delhi 110020, India

© The Author(s). 2021 **Open Access** This article is licensed under a Creative Commons Attribution 4.0 International License, which permits use, sharing, adaptation, distribution and reproduction in any medium or format, as long as you give appropriate credit to the original author(s) and the source, provide a link to the Creative Commons licence, and indicate if changes were made. The images or other third party material in this article are included in the article's Creative Commons licence, unless indicated otherwise in a credit line to the material. If material is not included in the article's Creative Commons licence and your intended use is not permitted by statutory regulation or exceeds the permitted use, you will need to obtain permission directly from the copyright holder. To view a copy of this licence, visit http://creativecommons.org/licenses/by/4.0/. The Creative Commons Public Domain Dedication waiver (http://creativecommons.org/publicdomain/zero/1.0/) applies to the data made available in this article, unless otherwise stated in a credit line to the data.

FIs have also been offered in a wide range of health care services to the clients for promoting uptake and maintaining adherence to services [4, 5]. For example, FIs in the form of $5 grocery coupons were used in antiretroviral therapy to maintain adherence and continue tuberculosis prophylaxis in the USA [6]. FIs were also used to promote uptake of HPV vaccinations among young adolescent girls in England [7], as well as to promote access and increase institutional deliveries (e.g. antenatal care) in India [8].

Incentives were introduced in the realm of family planning (FP) with an aim of exploring pragmatic solutions to control the growing population. The Government of India in 1952, launched the first ever-FP program emphasizing primarily on fertility-regulation and stabilizing the population at a level consistent with socioeconomic development with an overarching aim for improving access to maternal and child healthcare [9].

In an effort to encourage FP adoption, the Government of India has been implementing various centrally sponsored schemes that offer FI to providers as well as acceptors of certain types of FP services [10]. The first FI scheme was deployed in 1956, in Tamil Nadu state, where INR (Indian National Rupee) 30 was provided to male and female acceptors of sterilization, to compensate for the loss of wages and for the aftercare [10, 11]. A central FP board was formed in 1956, and an equivalent of around 10 million USD of funds were allocated towards the initiation of a truly nation-wide FP action program [12, 13].

Since then, numerous FI have been introduced in various healthcare programs either to increase the coverage or to promote the uptake. One such example is Janani Suraksha Yojana in India which has been effective in achieving high institutional delivery within a span of few years. This meant that FI has a potential to increase institutional delivery which also serves as access points for post-partum FP [14, 15].

In addition, FIs were also introduced for uptake of various FP methods such as intra uterine contraceptive device (IUCD), sterilization, oral contraceptive pills (OCPs), emergency contraceptive pills (ECPs), and some new FP methods for acceptors as well as healthcare providers in India [11, 16, 17]. The details of which are mentioned below in Table 1.

Whilst there is enough global literature available on the effects of FI in healthcare services, the evidence thus far on the role of FI on uptake of FP services and the provision FP methods is limited and mixed [18, 19]. In addition, there is growing ethical concern regarding provision of FI to providers, where providers may have been promotingFPmethods that were linked to higher incentives, thus violating free and informed choice [1, 3]. While incentives can be believed to deviate a women's choice from the norm of full voluntarism, they also increase the number of options open to an individual [10]. A study by Sunil T. et al., on FP in India found that the

Table 1 Financial incentives for FP methods in India (in INR)

	**Post-partum Intra Uterine Device (PPIUCD)**	**Post Abortion Intra uterine Device (PAIUCD) D**	**Antara**	**Post-abortion sterilization (PAS)**		**Postpartum sterilization (PPS)**		**Non Scalpel Vasectomy (NSV)**
				**Districts with TFR > =3**	**Districts with TFR < 3**	**Districts with TFR > =3**	**Districts with TFR < 3**	**High focus districts**
Provider (General Practitioner)	150	150	n/a	n/a	n/a	n/a	n/a	n/a
Accredited Social Healthcare Activist (ASHA)	150	150	100	300	200	300	400	200
Acceptors	300	300	100	2000	1400	3000	2200	1100
Drugs /dressings	n/a	n/a	n/a	100	100	100	100	50
Surgeon	n/a	n/a	n/a	200	150	325	200	100
Anesthesiologist	n/a	n/a	n/a	50	50	75	50	
Nurse / Auxillary Nurse Midwife (ANM)	n/a	n/a	n/a	40	20	50	50	15
Operation theatre Technician (OT Tech.)/helper	n/a	n/a	n/a	40	30	50	50	15
Clerk./ documentation	n/a	n/a	n/a	30	20	n/a	n/a	n/a
Refreshment	n/a	n/a	n/a	20	10	n/a	n/a	10
Miscellaneous	n/a	n/a	n/a	20	10	n/a	n/a	10

Source: India M. Schemes/Guidelines- FP: National Health Mission [Internet]. 2020 [cited 30 April 2020]. Available from: https://nhm.gov.in/index1.php?lang=1&level=3&sublinkid=963&lid=470
1 USD = 73.48 INR (as on 13th sept, 2020)

likelihood of adapting a FP method increased when FIs were provided to a socio-economically homogenous population [11]. However, the study also showed that motivational programs promoting contraception uptake and FP were more effective than cash incentives in improving the long-term use of temporary family planningFP methods [11].

While most non-FP services have a fixed incentive amount, there are varying amounts available for the uptake different FP methods (Table 1). Owing to the limited evidence on the role of incentives in opting for FP method within a resource-limited setting, as well as the lack of in-depth understanding of how providers and clients view and respond to the FI in India, we have conducted this qualitative study to understand the motivators for uptake of FP methods and the role of FIs in the uptake and provision of FP services.

Methodology

Study design

We conducted this qualitative descriptive study in October–November 2018. This qualitative descriptive approach have been widely used in qualitative studies across the globe [20] The study involved key informant interviews and in-depth interviews with participants from different stakeholder groups. The stakeholder group included clients (male and female) who have recently accepted a FP method linked to financial incentive, government health officials, members of development partners working in FP, FP service providers at public health facilities, and ASHA/ Sahiyas (women from village who work as field level health workers and are incentivized to motivate community members to attend public health facilities).

Study site and sampling

We conducted the study in the state of Jharkhand which is one of the high priority states of India with high total fertility rate (TFR is average number of children per woman) of 2.6 which is above the national average of 2.2 [21]. Thirty seven percent of state’[illegible]pulation lives below the line of poverty [21]. The state also witnesses high unmet need for contraception (18.4%) and spacing of children (9.0%) compare to national averages [21]. Only 37.5% women use any modern FP method, out of which female sterilization (31.1%) is the most preferred [21]. Every two out of five women are illiterate [21].

In Jharkhand, we purposively selected two districts: Chatra and Palamu, which differ from each other in terms of performances on key health and socioeconomic indices, to allow for maximum variation. Chatra is a district with TFR > 3 and is among relatively poor performing districts of Jharkhand in terms of health indicators [22], whereas Palamu is a district with TFR less than 3 and better health indicators [23]. Within each district, two blocks (sub district level administrative units) were selected. We selected one block that is located at proximity (within 10 kms of the district headquarters) and one remote to district headquarter.

Study participants and sample size

A total of 64 interviews were conducted, which included: 20 in-depth interviews with clients (male and female) who had recently availed incentive linked FP services, 11 key informant interviews with government health officials and members of development partners working in FP; 33 in-depth interviews with FP service providers of public health facilities and the ASHA/ Sahiyas working in facility’s catchment area. (Table 2).

We utilized a case study approach in the selection of clients and the ASHAs. We purposively chose those ASHAs who had motivated the clients included in our study, to take up the FP method. This enabled us to get critical insights into differences that exist between the perspectives of the clients and their motivators on the quality, process and decision making around adopting a specific method of family planning.

The Jhpiego program team, which has been providing technical assistance [illegible]P programs to both Government of Jharkhand and Government of India for more than 10 years, identified and recruited decision makers, development partners, service providers, and ASHAs/LHVs for the study. Potential participants from these stakeholder groups were recruited such that they have a minimum of 2 years of work experience in FP. The list of clients who availed FP services from public health facilities of the four blocks (of the two selected districts) in the last 2 years, was solicited from ASHAs or ANMs serving in the catchment area of the public health facilities.

Data tools and data collection

Separate semi structured interview guides were developed for each stakeholder group [Refer Additional file 1]. The questions were exploratory to allow perspectives of participants to emerge during the interviews. The questions for clients covered the following domains: socio economic context, knowledge of FP method, decision making process in opting for the FP method, experiences of using the method and related services, role and use of FIs in the uptake of the method and opinion on monetary versus non-monetary incentives for FP methods. The questions for ASHAs/ Sahiyas and health service providers covered domains like their role and capacities, processes followed by them for different FP methods, their perspectives on what motivates client to take up a FP method (including the role of FIs) as well as role and importance of FIs for themselves to provide FP services.

Table 2 Study participants across groups of stakeholders

Stakeholder group	Number of participants	Role with respect to family planning services
Acceptors or clients of FP services (Men/Women) with incentives:		Acceptors (2 years prior of study) of incentive based FP methods who were motivated by the19 ASHAs or Sahiyas included in the study.
i) Non Scalpel Vasectomy (NSV)	4	
ii) Female sterilization (FS)	8	
iii) Post-partum Intra uterine contraceptive devices (PPIUCD).	8	
Development partners	4	Members of Institutions /non –government organizations in Jharkhand which had been undertaking or supporting family planning programs for the past 2 years
Service providers	14	Doctors; nurses and auxiliary nurse midwives (ANMs) involved in providing FP services at public health facilities or platforms;
ASHAs or Sahiyas	19	Community health worker who serves as a link between health system and the community members and act as a informer and motivator for various health programs within her village.
Government officials:		
Decision makers at state level	2	
Decision makers at district level	5	Involved in management of family Planning.
Total	64	

For government health officials and development partners, the domains included factors which influence FP service provision and uptake, their perspective on role of FIs in service provision and uptake of FP services, alternatives to FIs and its potential impact as well as suggestions or recommendations for improving services. These domains were decided based on available published literature, findings of a dipstick study as well as consultations between the authors which was guided by their experience with the family planning programs in India. All tools were translated into Hindi which is the local language in Jharkhand.

Prior to conducting the study, a dipstick study was conducted in another block of Chatra district. The dipstick study involved interviews with acceptors of different family planning methods, health services providers, community health workers, members of development partners as well as district level FP program managers. The objective of the dipstick study was to inform the study design, identify all relevant participant groups for the study and inform the contents of the study tools.

Before initiating data collection, approvals were obtained from the administrative authorities of the two districts. Potential participants belonging to the stakeholder groups of government health officials, development partners, health service providers and motivators were given an explanation of the study and then recruited by Jhpiego program team. Those who agreed for participation were then contacted by the study team for conducting their interviews. ANMs or ASHAs identified the client and contacted them first to know about their interest in participating in the study. If they expressed interest, their details were passed on to the study team which then contacted them for conducting the interviews. The in-depth interviews or key informant interviews were conducted at a place and time chosen by the participant. For clients and ASHAs, the interviews were conducted at their homes or a health facility nearby, while for participants belonging to the other stakeholder groups, interviews were conducted at their work places. A written informed consent was obtained before the interview and only the participant and interviewer were allowed to be present during the course of interview. This ensured privacy and confidentiality, which enabled participants to speak freely about various issues that were discussed. None of the potential participants who were approached refused to participate and no repeat interviews were conducted. Before initiating the interviews, the interviewers made some general conversation with the participants to make them comfortable and build a rapport. All interviews were audio-recorded and the interviewers also made field notes after completion of each interview. On an average, the interviews took 40 min each.

Independent research consultants who conducted the interviews were graduates with prior experience (at least 5 years) of conducting in-depth interviews, specifically in FP related studies. They were all fluent in Hindi language. Female interviewers conducted the interviews with female clients while male interviewers conducted interviews with male clients. For participants belonging to other stakeholder groups, interviews were conducted by either male or female interviewers. Study team also included two experienced qualitative researchers, who transcribed and

translated the interviews from local language Hindi to English. Two co-authors (AS, AG), who were involved in supervising data collection, crosschecked each of the transcripts against the original recordings to ensure quality. The transcripts were not returned to participants for their comments or approval.

Data analysis

The analysis of data was done using Atlas ti 8. The two qualitative research experts coded the transcripts and analysed the data using content analysis in the style of the constant comparative method [24]. In this process Braun and Clarke's six step framework was followed [25]. This analysis blended both inductive and deductive strategies. A hierarchical coding structure was developed based on the open iterative coding of interview transcripts (to allow for inclusion of emergent codes) as well as the preidentified domains of interview guides. The two qualitative researchers coded random selections to check for reliability, inter-coder agreement as well as appropriateness of the coding scheme. Finally, they used the established codes to illustrate the thematic categories that result for each category of study participant. Both theme saturation as well as consensus between the two coders was achieved. Illustrative quotations in English linked to the participant category have been used in the results section. Feedback on analysis was not obtained from participants.

Results

1. Profile of the participants

Family planning acceptors or clients were married men and women aged between 18 and 40 years, having at least one child and residing in rural areas of the selected districts. The educational status ranged from being illiterate to college graduates. All women clients were housewives. All family planning acceptors belonged to lower, lower middle or middle income groups. ASHAs were women belonging to the village they served and their educational status ranged from completing high school to college graduation.

The other stakeholders which included service providers, development partners and decision makers were minimum graduates and had an experience of at least 2 years in implementation or service provision o[illegible]mily planning programs.

2. Uptake of FP methods

All stakeholders unanimously agreed that among limiting family planni[illegible]ethods, female sterilization was the most popular by[illegible] Women generally preferred this method once their family was complete. It was perceived as a simple "onetime" solution, with relatively lesser associated side effects or chance of complications. Male sterilization, on the other hand, was least popular of all the methods.

Among spacing methods, condoms, IUCDs and OCPs were the most preferred, although there were many myths or misconceptions linked to IUCDs and OCPs in the community. Uptake of spacing methods has been increasing over the past few years, although many still preferred traditional methods like calendar method or withdrawal method for spacing pregnancies. Injection depot-medroxyprogesterone acetate (DMPA) and Centchroman pills, which were recently introduced (2017) in the public health system, are yet not as popular due to lack of information about them among general public in the study districts.

3. Factors influencing the decision on uptake of FP method

Factors which emerged as key influencers of client decision to take up an FP method in the two study districts are following:

3.1. Felt need for a small family

Felt need for a small family emerged as the single most important driving factor for uptake of a family planning method in the two study districts. Almost all the clients who had opted for a limiting method stated that they took the decision as they had achieved their desired family size and did not wish to have more children. They cited increased costs associated with managing care and education of children and emphasized that smaller families are easier to manage with the means available. Participants from other stakeholder groups also identified the felt need for smaller families as the primary motivating factor behind couples' decision to adopt a permanent family planning method.

> "*Getting sterilization is good forever. It will be good as the number of children will be less. We will be able to feed them well, take good care of them and educate them well.*" – Female sterilization acceptor.

3.2. Health concerns

Almost all the PPIUCD clients, who accepted the method after their delivery, stated that they wanted to adequately space their subsequent pregnancies. They feared that frequent pregnancies would make them "weak" and "sick" and this would also adversely affect the health of their children. ASHAs or their motivators agreed that health concerns around closely spaced pregnancies was an important factor influencing clients to take up spacing methods like IUCDs, pills or condoms.

Majority of men who opted for NSV stated that they did so out of concern for the health of their wives. They

stated that they felt their wives were not healthy enough to undergo a surgery (female sterilization procedure) and therefore took the decision to undergo sterilization themselves.

> "*I had got two kids through C-section of my wife and that is why I thought that this operation (female sterilization) is risky for her. So I got it done*" - NSV client on why he decided to opt for the method.

3.3. Motivation by Sahiya/ASHAs

All health service providers, decision makers and development partners noted that motivation of clients by ASHAs played a very important role in uptake of family planning services. Majority of women clients reported that ASHA of her village was one of the primary sources of information on FP methods and that they always trusted her to give the correct information and guidance. ASHAs too stated that motivating clients to take up a method was their primary role with respect to family planning program and they had to follow up several times with some couples to motivate them to opt a method. They also reported the need to sometimes motivate family members of clients for ensuring that they take up the method of their choice.

> "*Sahiya didi (ASHA) told me that if I am really worried then I should have Copper T; there is no better effective method than this.*" – PPIUCD acceptor on why she decided to opt for the method.

3.4 Advise by family members, friends or neighbours

Many clients stated that their decision to opt for a family planning method was taken after consulting their partner, mother in law or any other family member who had used the method before. A few clients also mentioned taking advice of their neighbours and friends before opting for the method

> "*My husband told me. Then my mother-in-law and father-in-law also told me.*" – Female sterilization acceptor on why she decided to opt for the method.

3.5 Myths and misconceptions on methods

It emerged that there were many myths and misconceptions prevalent in the community regarding spacing methods like the IUCD and OC pills, which served as a deterrent to their uptake. A few common misconceptions associated with OC Pills were that they lead to weight gain or cause darkening of the skin. IUCDs on the other hand were believed to cause cancers or travel up into chest cavity and cause problems. With respect to NSV, there is a widely prevalent myth that it makes the man weak or less masculine, which may impair his ability to work and earn for the family and therefore even women don't want their husbands to take it up.

> "Why should my husband get NSV …. he is the bread-winner of the family …. if he becomes weak then what will happen to us" – A woman client who had undergone sterilization.

3.6 Asymmetric information on methods

It was also evident that counselling or information provided to clients on FP methods was inadequate. Although the ASHAs reported giving information to clients on all available choices, majority of the clients denied being informed about other available options during their decision making process. When probed, majority of the clients were not able to elaborate on the expected side effects of the methods they were currently using. However, the clients felt that their information base was sufficient for them to make an informed choice.

3.7 Other factors

Religious norms and preference for a male child also influenced the uptake of FP services. A couple of clients did state that they opted for female sterilization after having a male child, as after that they felt their family was complete. Few ASHAs and service providers pointed out that uptake of female sterilization was very low in tribal areas and Muslim dominated communities, owing to their cultural and religious norms.

4. Influence of financial incentives on uptake and provision of FP services

4.1 Influence of financial client's decision to opt for a FP method

All clients, including both women and men, categorically stated that their decision to opt for a family planning method was primarily driven by their felt need to limit or space subsequent births. They said that they would have opted for the method irrespective of whether government provided them with financial incentives or not.

> "*The government gives us (financial incentive) or not it doesn't matter to us. We have a desire to do NSV, so we go and get it done.*" – NSV Client.

Many clients did welcome the financial incentives though and said they felt happy about the amount that the government was providing. Most of them stated that they utilized the money to take good care of themselves by eating nutritious food and buying medicines, to help them recuperate from the surgery. A few clients said that the money helped them compensate for their travel expenses, wage losses or expenses incurred in availing these services at the public health facilities.

A couple of clients informed that they were not aware of any financial incentive attached with the method of their choice until after the procedure.

Majority of Sahiyas and service providers echoed the same sentiment that clients would avail FP services irrespective of financial incentives due to their felt need. However, few of them did state that financial incentives may be a primary driver for clients belonging to the lower socio economic strata of the society.

Decision makers and members of development partner agencies however attached more importance to financial incentives for clients and felt that they were important drivers of family planning uptake, especially for those from lower socio economic strata of the society.

> "*We belong to a district where there is lot of poverty, and I think this incentive people should get*"- District level official.

Couple of decision makers also suggested increasing the amount of financial incentives for clients, especially those attached with spacing methods, to improve uptake of FP methods in their area.

4.2 Influence of financial incentives on service provision by ASHAs or motivators

It was evident that financial incentives play a very key role in motivating ASHAs for performing their family planning program related duties. ASHAs are not salaried employees and therefore the financial incentives attached with their different duties are the primary source of income for them.

Majority of the ASHAs clearly stated that financial incentives attached to their FP related work are very important to them. Many of them were able to recall the exact amount they had received as incentives for family planning in the past 1 year. Few of them also emphasized that the amount given was not commensurate with the 'hard work' they put in and therefore should be increased. Some also expressed displeasure over delays in payment, which they felt was demotivating.

> "*We do this work and we like doing it. Our incentive money should also be released on-time. We do our work fully but if the amount is paid in time so we will do the work happily. We work everywhere and also want to do a better work, so that we get the cash rewards at a proper time interval*" – ASHA, Chatra district.

Participants from other stakeholder groups agreed that financial incentives served as a key motivation factor for Sahiyas to do their work. Some of them agreed with the demands of ASHAs for increasing the amount of financial incentive for them. They also emphasized on ensuring timely payment to them for keeping their motivation levels high.

A couple of service providers and members of development partners did raise concerns on selective promotion of FP methods, which have incentive amounts associated with them, by the ASHAs.

> "*ASHA is more motivated to give as much information as possible for these methods (attached to incentives).*" – Member of Development Partner.

4.3 Influence of financial incentives on service provision by nurses and doctors

It emerged that for doctors, nurses and ANMs, who are salaried employees, the financial incentives served as an extrinsic motivation for carrying out their family planning related work. It was perceived as a 'token of appreciation' from the government for their efforts. Majority stated that the amount was important to them, however they did add, that they would continue to provide their services irrespective of whether they received the incentive amount or not.

Participants from other stakeholder groups (government officials, development partners) agreed that financial incentives were important motivators for the nurses and doctors as well. A few of them also felt that different amount of incentive money for different methods does influence service provision by ANMs and nurses as they tend to 'push' those methods which have incentives associated with them.

5. Perspectives on discontinuing financial incentives

5.1 Discontinuing client incentives

There were mixed opinions on discontinuing financial incentives for clients. While all clients stated that they would have opted for the method irrespective of whether financial incentives were attached to them or not, some of them did emphasize that the incentive amount was also important for them. Few clients said that although the amount does not matter to them, but it may be important for the poorer clients.

Among the ASHAs, majority felt that discontinuing financial incentives for clients will adversely impact FP uptake to some extent, as there is a section of clients for whom incentive money is an important motivating factor. Remaining ASHAs, on the other hand felt that owing to the strong felt need for FP methods, discontinuing financial incentives for clients would not have any adverse effect.

Majority participants from the other stakeholder groups concurred that discontinuing incentives will adversely impact uptake of family planning services

especially among the poorer sections or among those living in remote locations. Government officials particularly were not in favor of discontinuing client incentives in one go. A government official stated that one needs to first study the impact by discontinuing incentives in a few districts and then come to a conclusion.

> "*A pilot project needs to be conducted to see what the effect in the region is once it is withdrawn and then compare it with other region. If all are withdrawn all of a sudden then there will be problem*" – A government official, Jharkhand.

5.2. Discontinuing incentives for ASHAs and service providers

Almost all Sahiyas clearly stated that financial incentives were their primary motivation to work. Many stated that unlike other service providers, they were not salaried employees and entirely depended on these financial incentives to run their families. Few also put forward their demand to increase the amount of incentives for them as it was not commensurate with the amount of effort they were putting in.

> "*We are working for the money only, either it is less or more. If we don't get money how will we work?* "– ASHA.

Nurses, doctors, government officials and development partners, all echoed the same sentiment and opined that FP services would be impacted majorly if incentives for ASHAs were scrapped. ASHAs played a key role in motivating clients and any step which de-motivates them would have major implication on the FP program.

ANMS, Nurses and doctors stated that discontinuing incentives for them would not affect the services that they provide as they were being paid (salary) to give their services.

Government officials and development partners though expressed their concern that scrapping incentives for service providers may adversely affect their motivation levels especially of nurses and junior doctors and therefore may have an adverse effect on the program. They emphasized that service providers receive financial incentives for their work across many health programs like maternal health, immunization etc. and scrapping incentives in the family planning program may demotivate them from actively giving services for the program.

> "*The doctor and the assistant get motivated when they get incentive because there is a series of incentives in all programs. Like in maternal health (MH) caesarean section, the doctor, nurse and even the 4th grade gets incentive. So if you give in some program and not in some, it will demotivate people*" - A district level government health official.

6. Perspectives on replacing financial incentives with non-monetary incentives

6.1. For clients

Many clients stated that they would be fine with any non-monetary incentive which the government offers them in place of cash like food items, clothes etc. Some did convey that they would prefer cash incentives as it would give them the freedom to buy things as per their needs. ASHAs too had a mixed opinion on the issue. Many of them felt that clients will appreciate non-monetary incentives more as they would get it right after accepting a method and would not have to wait like in case of financial incentives. A few of them stated that clients use the incentive amount for purchasing nutritious food items to help them recuperate better after surgery and therefore replacing financial incentives with nutritious food items would be a better option. A few ASHAs did however agree with the client who preferred financial incentives.

Service providers, government officials and development partners were more inclined towards continuing with financial incentives as non-monetary incentives may not meet every client's requirement. They also raised the issue of increased chance of corruption with disbursement of non-monetary items. Some government officials also pointed out the logistical challenges in distribution of non-financial incentives, which they felt would be a major barrier.

6.2. For ASHAs and service providers

For ASHAs, there was agreement among most respondents on the need for financial incentives, as they were not salaried and these financial incentives were their only source of income.

For ANMs, nurses and doctors, there were mixed opinions. While many service providers themselves stated that the 'token of appreciation', either monetary or non-monetary, would motivate them, government officials and development partners were more inclined on continuing with the financial incentives. They felt that non-financial incentives like certificates or rewards for good performance could be supplemented with monetary incentives. However, they were not in favor of completely scrapping monetary incentives as they felt it was important for their continued motivation.

> "*It will impact, but not their pro-activeness as monetary would. Though "recognition" matters but it doesn't give a push like money*" – Member of a development partner on whether non-monetary incentives would motivate service providers.

7. Issues with disbursement of financial incentives

Delay in payment of incentives was reported by some clients and ASHAs. Many participants emphasized on the need to ensure early payment of incentives, especially for the ASHAs and the clients, for keeping their motivation levels high.

ASHAs highlighted the importance of timely payment to the clients stating that delays adversely affect their relationship with their clients who then tend to lose their trust in them. This, they said hampers their work as people in her area start doubting her intentions and words.

> *"So then sometimes beneficiary starts talking and doubting us, asking what is the reason that Sahiya (ASHA) is not making us get our incentives."*
> – ASHA.

Government officials acknowledged that delays do happen in certain cases when the clients do not have a bank account or the documentation isn't complete from their end. They however did point out that such instances are now reducing as more and more clients are getting their bank accounts opened. They stated that the primary reason for this delay is at the client level (documentation issues), temporary unavailability of funds and lack of human resources.

Discussion

In this study, we described the following: uptake of various FP methods, factors influencing uptake of FP methods and the role of FI in family planning services in Jharkhand state of India, through qualitative interviews with acceptors of FP methods linked with FI, various cadre of health care providers, government health officials and development partners.

The key themes emerged from our study are:

- firstly, FI are not a primary factor influencing uptake of FP methods however FI are welcomed by clients as it is supported their recovery and covering travel expenses; since some of the respondents mentioned delays in receiving FI, non FI may also be important to consider.
- Secondly, since ASHAs / Sahiyas are not salaried employees, FI is the source of income for them, however there is a risk of promoting FP methods linked with higher FIs.
- Thirdly, removing FI or providing no FI for other health care providers (doctors, nurses) may be suitable option.

Amongst the ASHAs and clients sampled in the study female sterilization was reported as the most popular limiting method, the findings are suggestive of similarity with the results from nationally representative study conducted in 2015–16 where female sterilization was used by 36% of currently married women in India [21]. This popularity could be due to convenience, free provisioning at public health centers, and provision of compensation [26] and myths around temporary FP methods [27]. In our study women were against their husband undergoing vasectomy, due myth that would make them weak. However, a study conducted in Punjab state of India revealed that the main concern of the men was that if they get it done and their partners conceives then it would bring a bad name to the woman as well as to the family [28]. This difference might be due to primarily two reasons: differences in state and population interviewed. The participants in the study conducted in Punjab state were graduates and post-graduates; while in our study we had mixed population in terms of educational level. Moreover, these two states differ by socio-demographic characteristics, education, infrastructure and overall development. Our result is in consistency with other studies conducted across India, that highlighted various myths and misconceptions around NSV, including fear of surgical procedure; fear of post-operative weakness, inability to function sexually, failure of NSV and lack of knowledge related to NSV [29–33]. Similarities between our results and results of these studies shows that myths and misconceptions around male sterilization did not change over the years, implying these misconceptions are deep-rooted in the communities that requires innovative approaches to address it. The studies around the world suggested that the myths and misconceptions around NSV can be addressed by improving male involvement (especially men's interest in, knowledge of, and participation in FP), and encouraging their dialogue with service providers [34].

As per clients in our study, FIs did not act as a motivator for accepting FP method. This result is in line with qualitative study conducted in West Bengal, where female FP acceptors noted that they would opt for sterilization procedure even in the absence of FI; however, all respondents emphasized the need of having at least one son for the family progression and care provider to them during the old age [35]. A review conducted by Heil and colleagues [10] concluded that FP behaviours, like other behaviours, are sensitive to incentives and recommended that future research is needed as there is a lack of evidence based on rigorous studies. Additionally, a study conducted in Mexico [36] on the effect of conditional cash transfer program concluded that cash transfer was positively associated with

increased uptake of modern FP methods by adolescent and young adult women. However, it didn't show direct effect on adolescent pregnancy.

Several, studies conducted in India [29, 30, 37] concluded that FI are one of motivational factors for men to accept vasectomy, along with word of mouth. However, our result revealed that FIs are not a motivator for accepting NSV, the similar result was found in a study conducted in Andhra Pradesh state of India where FI were not significantly associated with acceptance of NSV [38].

In our study the perceived need for a small family, health concerns, motivation by Sahiyas/ASHAs, advice by family members, friends and neighbours', religious norms and son preference emerged as key motivational factors for uptake of FP methods. Similar results were found by Scott and colleagues in Uttar Pradesh, India where respondents mentioned a desire to limit number of children and educate children well [36] as a primary reason for accepting FP methods. However, despite of a clear preference for small families, the desire for male children may result in increased family size, with women continuing to bear children until either they give birth to a son or until some adverse event occurs [30, 35]. Furthermore, studies found that certain religious norms also prevented acceptance of sterilization services [36]. The majority of men in our study opted for NSV because the concern related to the health of their wives. Similar results were shown by Shattuck and colleagues who cited concern for women's health (desire to avoid pregnancies, births, and contraceptive side effects) along with desire to limit births, limited financial resources and dissatisfaction with other FP methods as reasons for acceptance of NSV globally [37].

The results of studies exploring the role of conditional cash transfer in increasing institutional deliveries [39, 40] revealed that FI is not a key motivating factor to access institutional delivery; rather support from community workers, growing individual perception of the importance of "safe" and "easy" delivery was an enabling factor to access institutional delivery services. Similarly, a study conducted in Turkey and Latin America [41] emphasized that beliefs around traditional and modern biomedical practices, sociocultural norms, gender relations, and the quotidian experience of poverty in many dimensions play role in health seeking behaviour rather than conditional cash transfer.

Although some of the respondents in our study welcomed FI and used it for covering travel expenses and getting nutritious food after surgery, some of the clients were also in favour of getting non FI like clothes, food items; however, providers expressed concerns with replacing FI with non FI as it may not meet every clients' needs and will have some logistical challenges. Opposite to our results, a randomized experiment conducted in Ecuador reported that respondents were in favour of FI over vouchers, food items due to autonomy that comes with FI [42].

The government officials and development partners in our study emphasized the importance of FI for lower socio-economic group of the society. The impact evaluation of a large conditional cash transfer program aiming at increasing institutional delivery in India found improved rates of institutional deliveries, however poorest women didn't benefit from the program [43]. On the other hand, an evaluation of cash transfer programs in Latin America found that cash transfer programs are effective in improving preventive health care, and raising household consumption among poor households [44]. Although there is no literature on how FI can impact specifically uptake of FP services, an evidence suggests that population with lower income are likely to be more responsive to FI in general [45]. On the other hand, Voigt concluded that uptake of FI linked with health promotion is unequal, with more affluent groups more likely to take advantage of the benefits [46]. Moreover, to our knowledge there is no research on impact of FI is removal on FP services update, studies on smoking cessation [47], drug abuse [48], retention in HIV prevention and treatment [49] suggests that change in response to FI return to baseline, once FIs are discontinued. Additionally, a systematic review on the effectiveness of FI on health behaviors such as smoking cessation, diet, alcohol consumption and physical activity suggested that positive effect might last for few months beyond the provision of FI [50]. Hence, there is a need for a study to better understand impact of removing FI on uptake of FP services, specifically for lower seriocomic group of population.

Our study found that FI were major motivator for Sahiyas /ASHAs, the similar findings were highlighted by another qualitative study conducted with ASHAs and their families in two districts of India [51]. Likewise, FI were identified as a major motivating factor in a study conducted in five states in India [52]. Our studied revealed the issues around the low rate of incentives for ASHAs/ Sahiyas and delayed payment. These results corroborate with studies conducted across India where low rates of FI relative to the level of efforts required to complete their responsibilities, magnified by delayed and incomplete payments was cause of dissatisfaction among ASHAs and their families [51–56].

Our study highlighted the possibility of bias among ASHAs/ Sahiyas towards FP methods linked with higher incentives. This was indicative as many acceptors of FP methods in our study mentioned that they were only informed about the method they had adopted during their interaction with ASHA/ Sahiyas. While ASHAs/Sahyas, stated having briefed clients about different FP methods,

however, they also highlighted IUCD and sterilization as the major FP methods which were linked with higher incentives. Government officials and development partners also expressed their concern about the possibility of biased counselling because of differential incentives linked to FP services. A study conducted in Bangladesh revealed that even though FI is successful in increasing FP use, there is a potential for bias as FP motivators received FI for referring clients for sterilization [57]. Furthermore, it was noted that FP motivators while referring clients for sterilization targeted low income group of society who might accept it because of FI [58]. A study conducted in India concluded that [51] ASHAs may have been prioritizing and promoting services linked to higher incentives such as sterilization.

Moreover, it is important to emphasize that a review on performance-based incentives in community-based FP programs showed that differential amount of FI has a potential to steer clients towards one method, thus breaching the principle of informed choice [19].

Additionally, several studies have mentioned biased counselling as violation of free and informed choice [53, 54]. On the other hand, ASHAs may have been focusing on sections of the community that are easier to persuade, leaving behind more vulnerable group of population [53].

Our study found that FI were not important to other cadre of health care providers. But existing literature has mixed evidence on this, thus a study conducted among government employed medical officers in three states of India, found FI as one of the motivator along with job security, opportunities for promotion, working condition [59]. Another qualitative study conducted in two states of India among various cadre of health workers found that FI had limited motivating factor, especially when the amount of FI is nominal [60]. On the contrary a study conducted among health care workers in Pacific and Asian countries concluded that FI is an important motivating factor, especially in countries where salaries of health workers are low [61]. Our study results and other similar studies imply the need to reconsider other ways of motivating various health cadres considering Indian health care system.

Given that India's FP program has evolved and made several gains over the past few decades, and the fact that FI for both FP clients and service providers still constitute a major part of resources invested in India's FP program [62], there is a need for more in-depth understanding of the role FI plays in enhancing the provision and uptake of FP services in current context. India spent 85% of its total family planning expenditure on female sterilization, 2% on spacing methods and 13% on family planning-related activities such as procurement of equipment, transportation, Information Education and Communication (IEC) and staff expenses in 2016–17 [63]. Moreover, 93 and 87% of the total budget for terminal and spacing methods respectively went toward incentives payment in 2016–17 [62, 63].

Strengths and limitations

To best of our knowledge, our study is the first study which explores the perspective of male and female clients who opted for FP method, sahiyas, service providers, decision makers at the district and state levels and development partners on FI.

Thus, explores an opportunity to understand the role of FI in family planning from various stakeholders' experiences. Second, this study is a part of a larger program whose focus was to incentivize the quality of FP services. Therefore, we had the professional relationships with the different stakeholders at each level, that helped us in getting more candid results. Third, we have followed a case study approach in which we interviewed the same sahiya[illegible] associated with the clients helping us in understanding the link between the counselling and informed choice among clients. This helped in triangulation of data and enabled us to gain insights in the decision-making process of the clients and identify the key factors within the service provision system, which influenced their decision.

Our study had few limitations as well. Firstly, we did not interview women and men who had not accepted family planning methods despite counselling from ASHA/ Sahiyas and their perceptions on incentives. In addition, there is a possibility of sampling bias because we did not interview unmarried women. It would have been very difficult to recruit participants from this group of respondents, as they are very less likely not open up regarding their FP use, as culturally it is considered inappropriate for unmarried women to indulge in sexual activities. We also acknowledge that the working or non-working status of female clients was not considered while recruiting them in the study, and this may have an influence on their ability to access FP services. Secondly, due to limited number of men who had undergone vasectomy in the last 2 years, we could include only 4 men in our study who had undergone vasectomy. Thirdly, due to nature of few questions (around importance of financial incentives), respondents, especially client and ASHAs, may have provided socially desirable responses to them, although all efforts were made for eliciting candid responses during the interviews.

Recommendations

In light of the findings from our study we propose the following recommendations: firstly, there is a potential to redesign the ways FIs are delivered and pilot innovative solutions. This could be done by continuing

incentivizing ASHAs/ Sahiyas for motivating people to get FP services; discontinuing FIs for senior doctors as senior doctors considered amount as token amount for appreciation; FI for senior doctors can be substituted by facility based FIs based on pre-defined quality standards Moreover, uniform incentives to ASHAS for all FP methods can minimize any possibility of bias towards methods with higher incentives.

Furthermore, setting up robust monitoring on ASHAs/ Sahiyas activities linked to incentives, would help to timely identification and appropriate action against promotion of services linked to higher FI. Additionally, it is imperative to ensure timely payments of incentives to both the providers and clients. Considering digital technology penetration across India, online / application-based integrated system for clients, ASHAs/Sahiyas and other health providers should be considered for real time tracking of incentive payments.

Secondly, as many respondents in our study mentioned the importance of FI for clients from lower socio-economic, it is crucial to continue client incentive only for the clients from lower socioeconomic groups. On the other hand, there is a need to explore non-FI for other categories of clients. Thirdly, it is crucial to address myths and misconceptions around IUCD, male sterilization. This could be achieved by couple counselling or Gender Role Models.

The above-mentioned recommendations can be piloted in a district or relatively smaller administrative area to see the effect of discontinuity of FI and replacement with non-FI, specifically for senior doctors and clients living above the line of poverty, on FP services. Also, it would be important to explore the role of different amount of FI for different FP methods acceptance as to our knowledge this area is not yet explored.

Conclusion

While FI while were not motivating factor for clients for accepting FP method, it was welcomed by clients and was used covering travel expenses and for buying nutritious food. FI were important motivating factor for Sahiyas /ASHAs as they are not salaried employees; however, they also reported dissatisfaction and concerns with remuneration amount that was not commensurate with the workload, delays in payments were concerns expressed by ASHAs/ Sahiyas. However, FI were not important for other group of health care providers. Future research will be required to explore the effect of FI on whether ASHAs /Sahiyas promote FP methods linked with higher FI; whether ASHAs/Sahiyas target specific groups of the community, hereby leaving the most vulnerable population; discontinuity of incentives for senior doctors & clients from better socio-economic status and replacement of FI with non FI for FP services.

Abbreviations

ANM: Auxiliary Nurse Midwife; ASHA: Accredited Social Health Activists; BMGF: Bill and Melinda Gates Foundation; TFR: Total fertility rate; CHW: Community Health Workers; ECP: Emergency Contraceptive Pill; FI: Financial Incentive; FP: Family Planning; IDI: In Depth Interview; INR: Indian National Rupee; IRB: Institutional Review Board; KII: Key Informant Interview; LHV: Lady Health Visitor; LMIC: Low and middle-income countries; MPV: Mission Parivar Vikas; NSV: Non-Scalpel Vasectomy; OCP: Oral Contraceptive Pills; PAIUCD: Post abortion intra uterine contraceptive device; PAS: Post abortion sterilization; PPIUCD: Post-partum intra uterine contraceptive device; PPS: Postpartum sterilization; QFP: Quality in Family planning; SDG: Sustainable development goals; USD: United States Dollar

Supplementary Information

The online version contains supplementary material available at https://doi.org/10.1186/s12913-021-06799-1.

Additional file 1.

Acknowledgements

We are most grateful and extend our sincere thanks to all people who participated in this study. We would like to thank our internal reviewer Young-Mi Kim for critically reviewing the manuscript and providing her valuable inputs. We would also like to acknowledge Udita Das and Mallika Mitra Biswas for their support in compiling the information from in depth interviews. We further thank our funder for their continued support.

Authors' contributions

AS, BS KL contributed to the concept and design of the study. AS, GU and AG were involved in study database creation, checking, and analysis. AG, GU, AS, AM helped draft the manuscript and BS contributed in interpretation of results, recommendation and conclusion section. All the authors contributed in finalizing the manuscript. KL is the guarantor for the project. All authors had full access to all of the data (including statistical reports and tables) in the study and can take responsibility for the integrity of the data and the accuracy of the data analysis. All authors read and approved the final manuscript.

Funding

The present study was funded by Bill and Melinda Gates Foundation (BMGF). The funding agency has no role in the study design, collection of data, analysis or report writing.

Availability of data and materials

Data are available from Jhpiego's internal institutional data access committee for researchers who meet the criteria for access to data. Data is under the custody of lead author. Any request for data sharing may be sent to lead author at his email address: 'kamlesh.lalchandani@jhpiego.org'. Data will be shared to interested researchers subject to approval from Jhpiego ICO.

Declarations

Ethics approval and consent to participate

The ethical approval was obtained from the Institutional Review Board (IRB) of Johns Hopkins School of Public Health (IRB No: 9114), USA as well as Sigma IRB, New Delhi, India- which is the local ethics committee. Written informed consents were taken from all the participants of the study.

Consent for publication

Not Applicable.

Competing interests

The authors declare that they have no competing interests.

Received: 12 October 2020 Accepted: 19 July 2021

References

1. Flodgren G, Eccles M, Shepperd S, Scott A, Parmelli E, Beyer F. An overview of reviews evaluating the effectiveness of financial incentives in changing healthcare professional behaviours and patient outcomes. Cochrane Database Syst Rev. 2011;7:3.
2. Eijkenaar F. Pay for performance in health care: an international overview of initiatives. Med Care Res Rev. 2012;69(3):251–76.
3. Abduljawad A, Al-Assaf AF. Incentives for better performance in health care. Sultan Qaboos Univ Med J. 2011;11(2):201.
4. Pantoja T, et al. Implementation strategies for health systems in low-income countries: an overview of systematic reviews. Cochrane Database Syst Rev. 2017;9:14-15.
5. Abdullahi LH, Kagina BM, Ndze VN, Hussey GD, Wiysonge CS. Improving vaccination uptake among adolescents. Cochrane Database Syst Rev. 2020;1:63.
6. Bock NN, Sales RM, Rogers T, DeVoe B. A spoonful of sugar...: improving adherence to tuberculosis treatment using financial incentives [Notes from the Field]. Int J Tuberculosis Lung Dis. 2001;5(1):96–8.
7. Mantzari E, Vogt F, Marteau TM. Financial incentives for increasing uptake of HPV vaccinations: a randomized controlled trial. Health Psychol. 2015 Feb;34(2):160.
8. Lahariya C. Cash incentives for institutional delivery: linking with antenatal and post natal care may ensure 'continuum of care'in India. Indian J Community Med. 2009 Jan;34(1):15.
9. National Health Portal. Centre for Health Informatics. Ministry of Health and Family Welfare (MoHFW), Government of India. National Programme for Family Planning. [Internet] [cited on 13th Sept, 2020]. Available at https://www.nhp.gov.in/national-programme-for-family-planning_pg.
10. Heil SH, Gaalema DE, Herrmann ES. Incentives to promote family planning. Prev Med. 2012;55:S106–12.
11. Sunil T, Pillai V, Pandey A. Do incentives matter? – Evaluation of a family planning program in India. Popul Res Policy Rev [Internet]. 1999;18(6):563–77 [cited 24 September 2019]. Available from: https://link.springer.com/article/10.1023/A:1006386010561.
12. Visaria L, Jejeebhoy S, Merrick T. From family planning to reproductive health: challenges facing India. Int Fam Plan Perspect. 1999;1:S44–9.
13. Simon JL. Income, wealth, and their distribution as policy tools in fertility control. In: Population and Development: The Search for Selective Interventions. Baltimore: Johns Hopkins University Press; 1976. p. 36–76.
14. Dongre AA. Effect of monetary incentives on institutional deliveries: Evidence from the Janani Suraksha Yojna in India. 2010. Available at SSRN: https://ssrn.com/abstract=1697105 or doi: https://doi.org/10.2139/ssrn.1697105.
15. Gupta SK, Pal DK, Tiwari R, Garg R, Shrivastava AK, Sarawagi R. Impact of Janani Suraksha Yojana on institutional delivery rate and maternal morbidity and mortality: an observational study in India. J Health Popul Nutr. 2012;30(4):464.
16. Family Planning [Internet]. Mohfw.gov.in. 2019 [cited 24 September 2019]. Available from: https://mohfw.gov.in/sites/default/files/06Chapter.pdf/
17. Ministry of Health and Family Welfare. National Health Mission. Schemes/Guidelines- FP [Internet]. [cited 24 September 2019]. Available from: https://nhm.gov.in/index1.php?lang=1&level=3&sublinkid=963&lid=470
18. Tessema G, Streak Gomersall J, Mahmood M, Laurence C. Factors determining quality of Care in Family Planning Services in Africa: a systematic review of mixed evidence. PLoS One. 2016;11(11):e0165627.
19. Bellows NM, Askew I, Bellows B. Review of performance-based incentives in community-based family planning programmes. J Fam Plann Reprod Health Care. 2015;41(2):146–51.
20. Kim H, Sefcik JS, Bradway C. Characteristics of Qualitative Descriptive Studies: A Systematic Review. Res Nurs Health. 2017;40(1):23–42. https://doi.org/10.1002/nur.21768 Epub 2016 Sep 30. PMID: 27686751; PMCID: PMC5225027.
21. International Institute for Population Sciences (IIPS) and ICF. National Family Health Survey (NFHS-4), 2015–16: India. Mumbai: IIPS. 2017.
22. Government of India,Ministry of Health and Family Welfare. District Factsheet: Chatra. Available at: http://rchiips.org/nfhs/FCTS/JH/JH_Factsheet_347_Chatra.pdf (accessed April 16, 2021).
23. Government of India. Ministry of Health and Family Welfare. District Factsheet: Palamu. Available at: http://rchiips.org/nfhs/FCTS/JH/JH_Factsheet_358_Palamu.pdf (accessed April 16, 2021).
24. Boeije H. A Purposeful Approach to the Constant Comparative Method in the Analysis of Qualitative Interviews, Quality & Quantity. Int J Met. 2002;36(4):391–409.
25. Braun V, Clarke V. Using thematic analysis in psychology. Qual Res Psychol. 2006;3:77–101. https://doi.org/10.1191/1478088706qp063oa.
26. De Oliveira IT, Dias JG, Padmadas SS. Dominance of sterilization and alternative choices of contraception in India: an appraisal of the socioeconomic impact. PLoS One. 2014;9(1):e86654.
27. Santhya KG. "changing family planning scenario in India: an overview of recent evidence," South & East Asia Regional Working Paper no. 17. New Delhi: Population Council; 2003.
28. Sood A, Pahwa P. Vasectomy: a study of attitudes, beliefs, knowledge and practice among literate men in Punjab, India. Int J Reprod Contracept Obstet Gynecol. 2014;3(2):418–23.
29. Zafer A, Gaur K, Kewalramani S, Gupta R, Manohar RK. Study of vasectomy adopters with special reference to motivational factors. Int J Sci Res Publ. 2013;3(9):1–6.
30. Scott B, Alam D, Raman S. Factors affecting acceptance of vasectomy in Uttar Pradesh: Insights from community-based, participatory qualitative research. The RESPOND Project Study Series: Contributions to Global Knowledge—Report No, vol. 3; 2011.
31. Shafi S, Mohan U. Perception of family planning and reasons for low acceptance of NSV among married males of urban slums of Lucknow city - a community based study. J Fam Med Prim Care. 2020;9(1):303–9.
32. Shattuck D, Perry B, Packer C, Chin QD. A review of 10 years of vasectomy programming and research in low-resource settings. Glob Health Sci Pract. 2016;4(4):647–60.
33. Lakshmi G, Chandrasekhran PA, RadhaRani G. A study on factors influencing the choice of permanent method of family planning. IOSR J Dental Med Sci. 2015;14(6):16–22.
34. Perry B, Packer C, Chin Quee D, Zan T, Dulli L, Shattuck D. Recent experience and lessons learned in vasectomy programming in low-resource settings: a document review. Durham, NC: FHI 360 and Washington, DC: The Population Council, the Evidence Project; 2016.
35. Kalra R, Phadnis S, Joshi A. Perceptual analysis of women on tubectomy and other family planning services: a qualitative study. Int J Reprod Contracept Obstet Gynecol. 2015;4(1):94–9.
36. Darney BG, Weaver MR, Sosa-Rubi SG, Walker D, Servan-Mori E, Prager S. The Oportunidades conditional cash transfer program effects on pregnancy and contraceptive use among young rural women in Mexico. Int Perspect Sex Reprod Health. 2013;39(4):205–14.
37. Murthy SR, Rao MD. An analysis of factors influencing the acceptability of vasectomy in Andhra Pradesh. Health Popul Perspect Issues. 2003;26:162–82.
38. Valsangkar S, Sai SK, Bele SD, Bodhare TN. Predictors of no-scalpel vasectomy acceptance in Karimnagar district, Andhra Pradesh. Indian J Urol. 2012;28(3):292–6.
39. Vellakkal S, Reddy H, Gupta A, Chandran A, Fledderjohann J, Stuckler D. A qualitative study of factors impacting accessing of institutional delivery care in the context of India's cash incentive program. Soc Sci Med. 2017;178:55–65 ISSN 0277–9536. https://doi.org/10.1016/j.socscimed.2017.01.059.
40. Sidney K, Tolhurst R, Jehan K, Diwan V, De Costa A. 'The money is important but all women anyway go to hospital for childbirth nowadays'-a qualitative exploration of why women participate in a conditional cash transfer program to promote institutional deliveries in Madhya Pradesh, India. BMC Pregnancy Childbirth. 2016;16(1):47. https://doi.org/10.1186/s12884-016-0834-y.
41. Adato M, Roopnaraine T, Becker E. Understanding use of health services in conditional cash transfer programs: insights from qualitative research in Latin America and Turkey. Soc Sci Med. 2011;72(12):1921–9. ISSN 0277-9536. https://doi.org/10.1016/j.socscimed.2010.09.032.
42. Hidrobo M, Hoddinott J, Peterman A, Margolies A, Moreira V. Cash, food, or vouchers? Evidence from a randomized experiment in northern Ecuador. J Dev Econ. 2014;107:144–15 ISSN 0304–3878. https://doi.org/10.1016/j.jdeveco.2013.11.009.
43. Lim SS, Dandona L, Hoisington JA, James SL, Hogan MC, Gakidou E. India's Janani Suraksha Yojana, a conditional cash transfer programme to increase births in health facilities: an impact evaluation. Lancet. 2010;375(9730):2009–23.

44. Rawlings LB, Rubio G. Evaluating the impact of conditional cash transfer programs: lessons from Latin America. Policy Research Working Paper; No. 3119. World Bank. 2003; [Internet] Available at: https://openknowledge.worldbank.org/handle/10986/18119 License: CC BY 3.0 IGO.
45. Dudley R, Tseng C, Bozic K, Smith W, Luft H. Consumer financial incentives: a decision guide for purchasers. Rockville, MD: Agency for Healthcare Research and Quality, AHRQ Publication No; 2007. p. 07(08)–0059.
46. Voigt K. Incentives, health promotion and equality. Health Econ Policy Law. 2012;7:263–83. https://doi.org/10.1017/S1744133110000277.
47. Volpp KG, Troxel AB, Pauly MV, et al. A randomized, controlled trial of financial incentives for smoking cessation. N Engl J Med. 2009;360:699–709.
48. Roll JM, Chermack ST, Chudzynski JE. Investigating the use of contingency management in the treatment of cocaine abuse among individuals with schizophrenia: a feasibility study. Psychiatry Res. 2004;125:61–4.
49. Yotebieng M, Thirumurthy H, Moracco KE, et al. Conditional cash transfers and uptake of and retention in prevention of mother-to-child HIV transmission care: a randomised controlled trial. Lancet HIV. 2016;3:e85–9.
50. Mantzari E, Vogt F, Shemilt I, et al. Personal financial incentives for changing habitual health-related behaviors: a systematic review and meta-analysis. Prev Med. 2015;75:75–85.
51. Sarin E, Lunsford SS, Sooden A, Rai S, Livesley N. The mixed nature of incentives for community health workers: lessons from a qualitative study in two districts in India. Front Public Health. 2016;4:38.
52. Bajpai N, Dholakia RH. Ravindra. Improving the performance of accredited social health activists in India (working paper no. 1). Mumbai: Columbia Global Centres South Asia; 2011.
53. Sharma R, Webster P, Bhattacharyya S. Factors affecting the performance of community health workers in India: a multi-stakeholder perspective. Glob Health Action. 2014 Dec 1;7(1):25352.
54. Bhatia K. Performance-based incentives of the ASHA scheme: stakeholders' perspectives. Econ Polit Wkly. 2014;55:145–51.
55. Bhatnagar R, Singh K, Bir T, Datta U, Raj S, Nandan D. An assessment of performance based incentive system for ASHA Sahyogini in Udaipur, Rajasthan. Indian J Public Health. 2009;53(3):166–70.
56. Saprii L, Richards E, Kokho P, Theobald S. Community health workers in rural India: analysing the opportunities and challenges Accredited Social Health Activists (ASHAs) face in realising their multiple roles. Hum Resour Health. 2015;13:95.
57. . Cleland J, Mauldin WP. The Promotion of Family Planning by Financial Payments: The Case of Bangladesh. Research Division, Working Papers 1990: No. 13. pdf.usaid.gov/pdf_ocs/PNABK863.pdf [accessed 6 October 2020].
58. Klitsch M. Sterilization decision is not unduly influenced by cash compensation. Int Fam Plan Perspect. 1990;16:156–67.
59. Purohit B, Bandyopadhyay T. Beyond job security and money: driving factors of motivation for government doctors in India. Hum Resour Health. 2014;12(1):12.
60. Rao KD, Ramani S, Murthy S, Hazarika I, Khandpur N, Chokshi M. Health Worker Attitudes Toward Rural Service in India: Results from Qualitative Research. Washington, DC: Health, Nutrition, and Population (HNP), International Bank for Reconstruction and Development [Internet]; 2010. [cited on 2 May, 2020]. Available at http://www-wds.worldbank.org/external/default/WDSContentServer/WDSP/IB/2010/11/23/000334955_20101123051207/Rendered/PDF/580190WP01PUBL1ral0service0in0India.pdf
61. Henderson L, Tulloch J. Incentives for retaining and motivating health workers in Pacific and Asian countries. Hum Resour Health. 2008;6(1):18.
62. Muttreja P, Singh S. Family planning in India: The way forward. Indian J Med Res. 2018;148 [Internet]. Available from: https://www.ncbi.nlm.nih.gov/pmc/articles/PMC6469373/.
63. National Health Mission. Government of India. Financial Management Report. New Delhi: Ministry of Health and Family Welfare. Government of: India; 2016-2017.

Publisher's Note

Springer Nature remains neutral with regard to jurisdictional claims in published maps and institutional affiliations.

Ready to submit your research? Choose BMC and benefit from:

- fast, convenient online submission
- thorough peer review by experienced researchers in your field
- rapid publication on acceptance
- support for research data, including large and complex data types
- gold Open Access which fosters wider collaboration and increased citations
- maximum visibility for your research: over 100M website views per year

At BMC, research is always in progress.

Learn more biomedcentral.com/submissions

BMC
